# Normalizing Plasma Renin Activity in Experimental Dilated Cardiomyopathy: Effects on Edema, Cachexia, and Survival

**DOI:** 10.3390/ijms20163886

**Published:** 2019-08-09

**Authors:** Ryan D. Sullivan, Radhika M. Mehta, Ranjana Tripathi, Inna P. Gladysheva, Guy L. Reed

**Affiliations:** 1 Department of Internal Medicine, University of Arizona College of Medicine–Phoenix, Phoenix, AZ 85004, USA; 2 Department of Comparative Medicine, University of Tennessee Health Science Center, Memphis, TN 38163, USA; 3 Department of Internal Medicine, University of Tennessee Health Science Center, Memphis, TN 38163, USA

**Keywords:** plasma renin activity, heart failure, aliskiren, dilated cardiomyopathy, neprilysin, edema, cachexia/sarcopenia

## Abstract

Heart failure (HF) patients frequently have elevated plasma renin activity. We examined the significance of elevated plasma renin activity in a translationally-relevant model of dilated cardiomyopathy (DCM), which replicates the progressive stages (A–D) of human HF. Female mice with DCM and elevated plasma renin activity concentrations were treated with a direct renin inhibitor (aliskiren) in a randomized, blinded fashion beginning at Stage B HF. By comparison to controls, aliskiren treatment normalized pathologically elevated plasma renin activity (*p* < 0.001) and neprilysin levels (*p* < 0.001), but did not significantly alter pathological changes in plasma aldosterone, angiotensin II, atrial natriuretic peptide, or corin levels. Aliskiren improved cardiac systolic function (ejection fraction, *p* < 0.05; cardiac output, *p* < 0.01) and significantly reduced the longitudinal development of edema (extracellular water, *p* < 0.0001), retarding the transition from Stage B to Stage C HF. The normalization of elevated plasma renin activity reduced the loss of body fat and lean mass (cachexia/sarcopenia), *p* < 0.001) and prolonged survival (*p* < 0.05). In summary, the normalization of plasma renin activity retards the progression of experimental HF by improving cardiac systolic function, reducing the development of systemic edema, cachexia/sarcopenia, and mortality. These data suggest that targeting pathologically elevated plasma renin activity may be beneficial in appropriately selected HF patients.

## 1. Introduction

Heart failure (HF) progression is affected by various pathways, including the sympathetic nervous system, the renin-angiotensin-aldosterone system (RAAS), and the natriuretic peptide (NP) system [1,2]. Activation of the RAAS is associated with left ventricular dysfunction, cardiac dilation sodium and extracellular fluid retention (edema) and cachexia/sarcopenia [3,4,5,6,7]. Current treatment guidelines [8,9] for HF broadly recommend therapeutics directed against angiotensin, aldosterone, or neprilysin, without the assessment of these biomarkers [5]. Nevertheless, it is increasingly recognized that there are disparities in treatment outcomes related to sex [4,7], race [10,11], geographic location [10], disease etiology, and genetic causes [12]. Analogous to cancer, HF has broad systemic effects on organs such as the lungs, kidneys, and liver, which are not emphasized by traditional classification systems [13,14]. Individual HF patients often have variable left ventricular (LV) function [15,16,17,18,19] and may progress at different clinical rates, and, thus, improving the treatment of HF may necessitate a precision medicine approach [20], which requires a two-pronged technique that relies on 1) identification of specific biomarkers, to enhance early diagnoses and etiologic classification and 2) a targeted treatment plan for the individual based on their specific profile.

Dilated cardiomyopathy (DCM) is a major cause of HF, which is associated with pathological dilation of the heart ventricles and a reduction in heart contractile or systolic function [8,9,12,21]. A well-established, translational mouse model of DCM with reduced ejection fraction (rEF) [22,23,24,25,26,27] fulfills the criteria of the American Heart Association Scientific Statement for Animal Models of Heart Failure [28]. We have previously reported that female mice with DCM develop HF at an accelerated rate, with worsening systolic function, increased atrial/B-type natriuretic peptides (ANP/BNP), lung edema, and reduced survival [27]. Prior to the development of Stage C HF, which is associated with edema and other biomarker abnormalities, female mice show increased plasma renin activity concentrations [27]. Renin is an enzyme that specifically catalyzes the first and rate-limiting step in the activation of angiotensin II (Ang II) and renin enhances the secretion of aldosterone, which promotes salt and water retention and worsens the development and progression of HFrEF [29,30,31,32]. Plasma renin activity increases with HF stages and varies between patient subsets [27,33]. Nevertheless, clinical studies (i.e., ASTRONAUT, ARIANA-CHF-RD, ATMOSPHERE) using direct renin inhibitors (DRI) have failed to improve outcomes in HF patients. However, these studies failed to select patients with pathological plasma renin activity levels, contained relatively few female patients, did not account for the confounding effects of other co-administered HF medications, used higher dose DRIs that affect renin as well as Ang II production, and commenced treatment late (e.g., initiation at Stage C rather than during Stage B) [34,35,36,37].

We examined the pathophysiologic contribution of elevated plasma renin activity concentrations to the progression of HFrEF as assessed by the development of edema, cachexia/sarcopenia, and mortality, in a randomized and blinded study. In contrast to previous trials, we intentionally chose an oral dose (100 mg/kg) of the DRI-aliskiren to selectively normalize pathological levels of plasma renin activity, rather than the larger doses, which may affect other pathways [38,39]. We show that targeted suppression with DRI-aliskiren, initiated at Stage B HF, reduces elevated plasma renin activity concentration to normal levels and decreased plasma neprilysin levels, without affecting the plasma levels of Ang II and aldosterone. The normalization of plasma renin activity was associated with improved heart systolic function, reduced edema, diminished cachexia/sarcopenia, and prolonged survival.

## 2. Results

### 2.1. Normalization of Elevated Plasma Renin Activity

**Figure 1:**
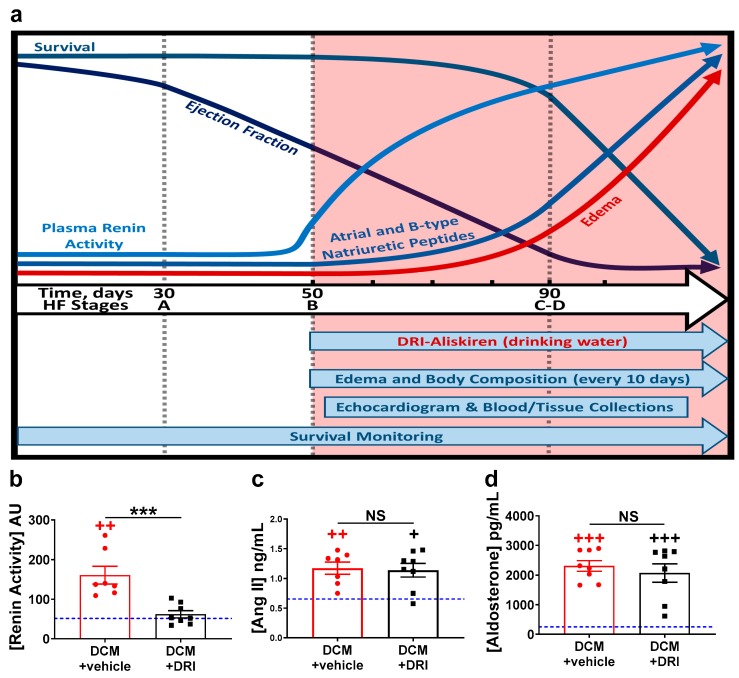
Heart failure stages, study design, and effects of aliskiren, a direct renin inhibitor (DRI). (**a**) Schematic overview of the natural history of heart failure (HF) progression, biomarker changes, and experimental design (salmon-colored area), in an established model of dilated cardiomyopathy (DCM) in female mice as reported [27]. Mice with DCM were randomly treated with aliskiren (DCM+DRI) or nothing (DCM+vehicle) in drinking water (see Methods Section). Black hash-marks indicate time points for measurement of body composition, while echocardiography and blood-tissue collection were completed at 90 days. The impact of aliskiren treatment on plasma (**b**) renin activity, (**c**) angiotensin II (Ang II), and (**d**) aldosterone levels at 90 days. The number of DCM mice is indicated. For reference, values for wild-type (WT) littermates are shown as a blue line (*n* = 4). Data analyzed with one-way ANOVA and represented as mean ± SE. Not significant (NS), ^++^ *p* < 0.01, ^+++^ *p* < 0.001 (red, WT vs. DCM+vehicle; black, WT vs. DCM+DRI), *** *p* < 0.001 (DCM+vehicle vs. DCM+DRI).

The effect of renin activity normalization was assessed in female mice with DCM as they pass progressively through the stages of HF development (Figure 1a) from Stage A (no HF), to Stage B (structural heart disease), through Stage C (edema, symptoms), Stage D (severe HF), and death [40]. As we previously reported, female mice with DCM begin to show declines in heart systolic function (ejection fraction) and increases in plasma renin activity concentrations around seven weeks of age (Stage B HF) [27], which precedes the development of progressive edema, further declines in systolic function, rises in ANP/BNP, and death as is typically seen in progressive HF (Figure 1a). Based on this data, female DCM littermates were randomly assigned to aliskiren treatment (DCM+DRI) vs. no treatment (DCM+vehicle) beginning at 50 days, Stage B HF. Three groups of female littermates were blindly examined: DCM control (DCM+vehicle), DCM+DRI and wild-type (WT) mice. When evaluated at 90 days, Stage C–D HF, treatment with the DRI significantly reduced elevated plasma renin activity concentration to normal levels as expected (*p* < 0.01, Figure 1b). Pathologically elevated plasma Ang II (Figure 1c) and aldosterone (Figure 1d) levels were not modulated by renin inhibition.

### 2.2. Normalization of Plasma Renin Activity Prolongs Survival and Delays Progression of Left Ventricular Systolic Dysfunction

**Figure 2:**
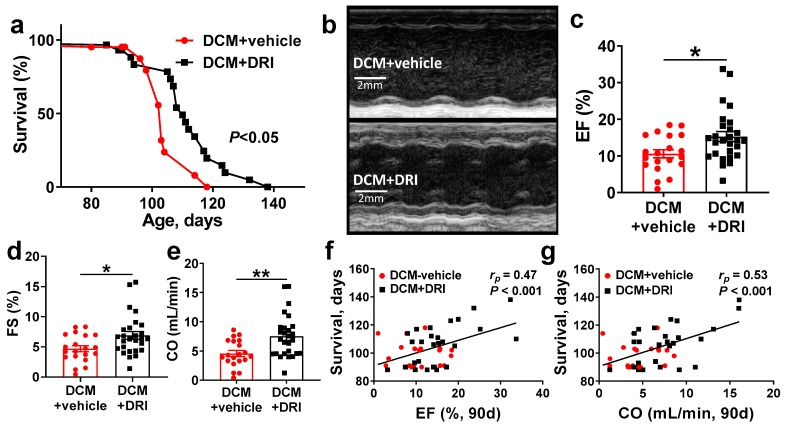
Direct renin inhibitor (DRI) treatment significantly improves survival and systolic function in mice with dilated cardiomyopathy (DCM). (**a**) Kaplan–Meier survival curves of control mice with DCM (DCM+vehicle, red, *n* = 13 deaths + 8 censored) vs. DCM mice treated with DRI (DCM+DRI, black, *n* = 21 deaths + 8 censored). WT (*n* = 4) values are provided for reference. (**b**) Short axis m-mode examples of DCM+vehicle and DCM+DRI treated mice at 90 days of age. (**c,d**) Left ventricular systolic function measured as ejection fraction (EF, WT = 62.8%) and fractional shortening (FS, WT = 34%). (**e**) Differences in cardiac output (CO, WT = 15.5 mL/min) between DCM+vehicle and DCM+DRI mice. (**f**) Pearson’s correlation analysis of 90-day EF and (**g**) CO vs. survival. DCM control mice (DCM+vehicle, red-circle, *n* = 20), DCM mice treated with DRI (DCM+DRI, black-square, *n* = 27). Differences between groups were analyzed by Mantel–Cox test and Mann–Whitney test. Pearson’s correlation coefficient (*r_p_*) and *p*-values are shown. Data are represented as mean ± SE, * *p* < 0.05, ** *p* < 0.01 (DCM+vehicle vs. DCM+DRI).

WT littermates had 100% survival throughout the 140-day study (data not presented). DCM+DRI mice significantly outlived the DCM+vehicle mice (median survival 110 vs. 103 days respectively, *p* < 0.05, Figure 2a). In the same experimental groups, cardiac structure and function were assessed by echocardiography at 90 days (Stage C HF with respect to DCM+vehicle group). Systolic function in DCM mice was improved with DRI treatment as assessed with m-mode imaging (Figure 2b) and measured by ejection fraction (EF%, *p* < 0.05, Figure 2c) and fractional shortening (FS%, *p* < 0.05, Figure 2d). Cardiac output (CO mL/min) was also improved with DRI treatment (*p* < 0.01, Figure 2e), reflecting changes in both heart rate (DCM+vehicle 419 ± 10 bpm vs. DCM+DRI 469 ± 14 bpm, *p* < 0.01) and changes in stroke volume (DCM+vehicle 11 ± 1 µL vs. DCM+DRI 16 ± 1 µL, *p* < 0.05). Contractile function assessed at 90 days by EF (*r_p_* = 0.47, *p* < 0.001, Figure 2f) and CO (*r_p_* = 0.53, *p* < 0.05, Figure 2g) were positively correlated with survival outcome.

### 2.3. Normalization of Plasma Renin Activity Delays Development of Systemic Edema and Cachexia/Sarcopenia

**Figure 3:**
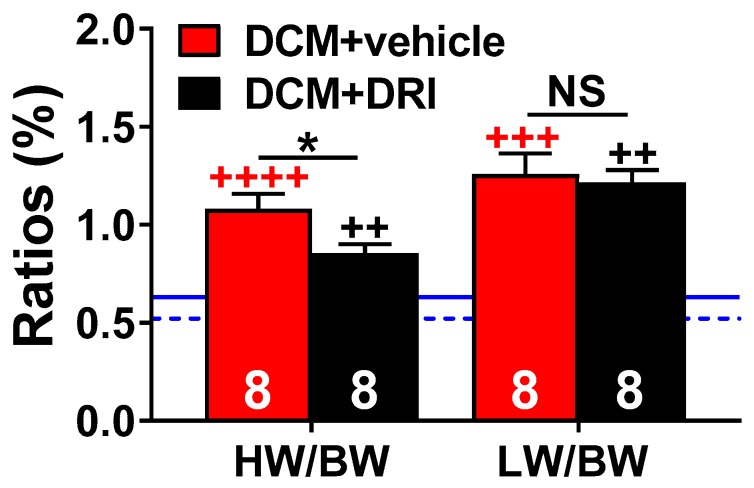
Morphometric changes at 90 days. Heart weight to body weight (HW/BW, %) and lung weight to body weight (LW/BW, %) ratios at a 90-day collection of censored subgroups. DCM control (DCM+vehicle) and aliskiren treated (DCM+DRI) group sizes are shown. For reference, the mean ratios for wild-type mice (WT, *n* = 4) for HW/BW are shown as a blue dashed line and the LW/BW as a solid blue solid line. Data were analyzed with two-tailed unpaired t-test and represented as mean ± SE. Not significant (NS), ^++^ *p* < 0.01, ^+++^ *p* < 0.001, ^++++^ *p* < 0.0001 (red, WT vs. DCM+vehicle; black, WT vs. DCM+DRI), * *p* < 0.05 (DCM+vehicle vs. DCM+DRI).

**Figure 4:**
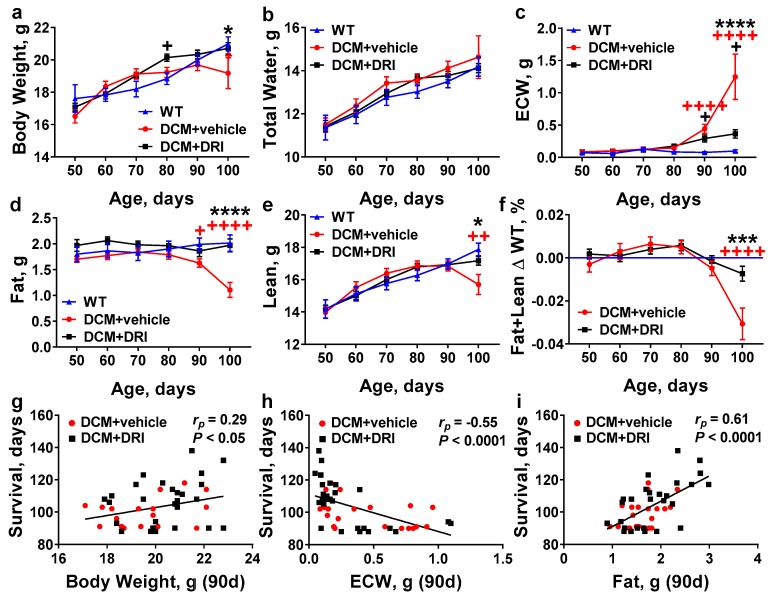
Effects of direct renin inhibition on systemic changes in mouse whole-body composition associated with heart failure progression. Age-related changes in (**a**) body weight, (**b**) total water, (**c**) extracellular water (ECW), (**d**) fat, (**e**) lean mass, and (**f**) combined fat and lean mass in DCM mice with (DCM+DRI) or without renin inhibition (DCM+vehicle) by comparison to wild-type (WT, without DCM) mice. WT (*n* = 9–15), DCM+vehicle (*n* = 10–27), DCM+DRI (*n* = 17–29). (**g**–**i**) Pearson’s correlation between survival (days) and (**g**) body weight; (**h**) extracellular water; and (**i**) fat measurements at 90 days. DCM+vehicle mice (*n* = 20) and DCM+DRI mice (*n* = 27). Differences between groups were analyzed by two-way ANOVA. Data are represented as the mean ± SE. Pearson’s correlation coefficient (*r_p_*) and *p*-values shown. ^+^ *p* < 0.05, ^++^ *p* < 0.01, ^++++^ *p* < 0.0001 (red, WT vs. DCM+vehicle; black, WT vs. DCM+DRI), * *p* < 0.05, *** *p* < 0.001, **** *p* < 0.0001 (DCM+vehicle vs. DCM+DRI).

To evaluate the effects of renin activity normalization, mouse hearts and lungs were examined at 90 days of age. Control (DCM+vehicle) mice had a significant increase in heart to bodyweight ratios (HW/BW) compared to WT (*p* < 0.0001, Figure 3) and DCM+DRI (*p* < 0.05, Figure 3) mice. DRI-treated mice had an increased HW/BW ratio compared to WT (*p* < 0.01, Figure 3). DRI treatment reduced the gross increase in cardiac weight of control (DCM+vehicle) mice by 20.4% (*p* < 0.05). Both DCM+vehicle and DCM+DRI groups were characterized by an increased lung weight to body weight (LW/BW) ratio compared to WT (*p* < 0.001 and *p* < 0.01 respectively, Figure 3). Body weights at 90 days were not significantly different between groups (Figure 4a); as a result, the ratio differences in HW/BW and LW/BW appeared due to the increased cardiac and lung weights of the vehicle and DCM+DRI mice.

Similar to humans with DCM, mice with DCM develop heart dilation, which progresses to symptomatic HF, water retention, and edema [24,26,27]. Edema is characterized not only by water retention in lung tissue (pulmonary and pleural), but also by systemic water accumulation in the cavities and/or tissues of the body (ascites and peripheral tissues). To quantify systemic water retention, we dynamically monitored body composition in all experimental groups every 10 days starting from 50 days of age, using quantitative magnetic resonance (QMR). Bodyweights were relatively consistent until 100 days when weights appeared to decline in DCM+vehicle controls compared to WT and DCM+DRI mice (*p* < 0.05 and *p* < 0.05, Figure 4a). Feed was provided to all mice ad-lib throughout the study; however, diet consumption was not specifically monitored. Total water measurements were similar among all groups throughout the study (Figure 4b). Systemic extracellular water (ECW, reported as free water in QMR measurements) began to rise after 80 days and became significantly elevated at 90 days of age in DCM+vehicle mice (*p* < 0.0001, Figure 4c). The progressive increase of ECW continued at 100 days in control (DCM+vehicle) mice compared to WT (*p* < 0.0001). Systemic ECW levels were significantly higher in DCM+vehicle vs. DCM+DRI mice when recorded at 100 days of age (*p* < 0.0001, Figure 4c).

The diagnosis of cardiac cachexia/sarcopenia in HF patients correlates strongly with an overall poor prognosis [41]. Similarly, the DCM mice developed measurable wasting as they progressed from late Stage C to terminal Stage D HF (Figure 4d–f). Body fat changed in the opposite pattern; treatment with DRI resulted in the maintenance of fat levels similar to WT through 100 days, whereas DCM+vehicle mice showed major fat losses by 100 days (*p* < 0.0001, Figure 4d). There was a significant decline in lean body mass in DCM control mice at 100 days, consistent with sarcopenia, by comparison to DCM+DRI and WT mice (Figure 4e). Overall there was significant wasting (Figure 4f) or cachexia, as measured by combined losses of fat and lean mass in DCM control mice by 100 days relative to WT (*p* < 0.0001) and DCM+DRI mice (*p* < 0.001).

Bodyweight at 90 days of age was marginally associated with survival (Figure 4g). A better measure was systemic ECW, which was negatively correlated with survival outcomes (*r_p_* = −0.55, *p* < 0.0001, Figure 4h). Fat measurements had the best correlation (*r_p_* = 0.61, *p* < 0.0001, Figure 4i) with survival. Lean changes (*r_p_* = 0.17, *p* = 0.2591) were not significantly correlated with survival.

### 2.4. Heart Failure Plasma Biomarkers Independently Respond to Direct Renin Inhibition.

**Figure 5:**
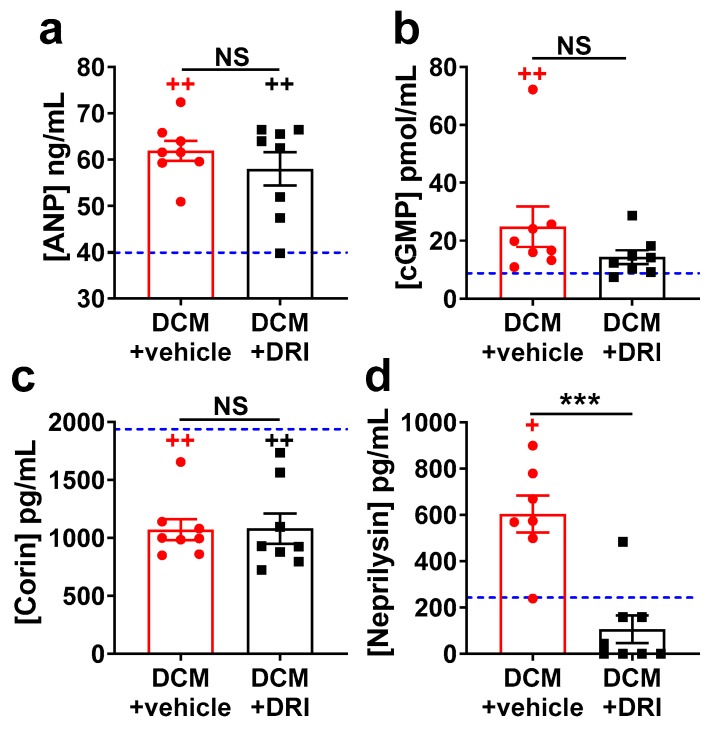
Impact of direct renin inhibitor, aliskiren (DRI-aliskiren) treatment on heart failure plasma biomarkers in female mice with dilated cardiomyopathy. (**a**) Atrial natriuretic peptide (ANP); (**b**) cyclic guanosine monophosphate (cGMP); (**c**) corin; and (**d**) neprilysin plasma levels. DCM+vehicle (red bar) and DRI-aliskiren treated (DCM+DRI, black bar) group numbers are indicated. For reference, values for wild-type (WT) mice are shown as a blue dashed line (*n* = 4). Data analyzed with one-way ANOVA and represented as mean ± SE. Not significant (NS), ^+^ *p* < 0.05, ^++^ *p* < 0.01 (red, WT vs. DCM+vehicle; black, WT vs. DCM+DRI) and *** *p* < 0.001 (DCM+vehicle vs. DCM+DRI).

HF biomarkers were measured in subgroups of mice at 90 days (Stage C/D HF). As expected, plasma ANP (*p* < 0.01, Figure 5a) and cyclic guanosine monophosphate (cGMP, *p* < 0.01, Figure 5b) levels were elevated, while plasma corin levels were reduced (*p* < 0.01, Figure 5c) in the DCM+vehicle group vs. WT littermates. ANP and corin plasma levels were not affected by DRI treatment (Figure 5a,c). Plasma cGMP levels in DCM+DRI group showed a non-significant trend toward WT levels (Figure 5b). Neprilysin levels were increased in DCM+vehicle mice compared to WT (*p* < 0.05, Figure 5d), consistent with human HF. DRI-aliskiren treatment, in DCM+DRI mice, normalized neprilysin to WT levels.

## 3. Discussion

HF has different etiologies and patients show differences in biomarker profiles. For example, expression and activation of the enzymes, hormones, and receptors of the RAAS are modulated by a variety of factors including sex [7,42]. Recent experimental studies have linked elevated plasma renin activity levels in female mice with DCM to accelerated, sex-related deterioration in systolic function, HF progression, and early mortality [27]. While active renin is acknowledged to initiate production of both Ang II and aldosterone [38,43], clinical studies have not rigorously examined plasma renin activity in the pathogenesis of HF [32,34,35,36,37,44]. Active renin concentration was recently suggested as a potential indicator for guiding HF with reduced EF (HFrEF) in addition to BNP and New York Heart Association classification [45]. The present data, in a translationally-relevant model of HFrEF [22,23,26,27], is the first evidence that targeted normalization of elevated plasma renin activity concentration by direct renin inhibition, at a dose that does not alter the Ang II-aldosterone axis, significantly prolongs life, preserves left ventricular function, reduces edema formation, and delays cachexia/sarcopenia.

There are limited experimental studies exploring the direct effect of renin activity inhibition in dilated cardiomyopathy [32]. In mouse models of acute myocardial infarction with ischemic cardiomyopathy [46] and diabetic cardiomyopathy [47], DRI treatment improved systolic dysfunction and prevented remodeling. Most rodent experiments dose aliskiren via osmotic pumps for subcutaneous administration at normotensive dosages (10–50 mg/kg SC ≈ 50–100% bioavailable = 5–25 mg/kg) [46,48,49,50]. In contrast, we elected to dose aliskiren orally (100 mg/kg ≈ 2.6% bioavailable = 2.6 mg/kg bioavailable) to mimic clinical administration and to take advantage of the known pharmacokinetics and pharmacodynamics of the drug as originally developed. This dose, as previously reported in mice [51], was effective in normalizing plasma renin activity (Figure 1b), but had no effect on Ang II levels between treated and untreated DCM groups. Although aliskiren plasma levels were not measured directly, the suppression of renin activity levels in plasma samples of DCM+DRI group compared to DCM+vehicle group supports aliskiren presence in circulation at 90 days of age, when plasma biomarkers were analyzed.

These experiments were performed in a well-characterized preclinical mouse model of DCM that is uniquely suited for the experimental re-evaluation of the role of plasma renin activity in the progression of HFrEF [22,23,24,25,26,27]. Mice with DCM pass through all stages of HFrEF with well-defined biomarker profiles, preservation of kidney function, and normal blood pressure through Stage D and death [26,27]. This animal model allowed for the investigation of DRI-aliskiren in DCM without clinical confounders (e.g., nutritional, environmental, concurrent or underlying disease status, and coadministration of additional medications). Female DCM mice were randomized to begin treatment at 50 days when pathologically elevated plasma renin activity concentration is first detected and there is an initial decline in EF corresponding to Stage B HF (Figure 1a) without the onset of edema and elevation of ANP/BNP plasma biomarkers [27,32]. DCM+DRI mice showed delayed development of Stage C HF as evidenced by the reduced ECW accumulation (edema) and a 7% increase in overall survival, which is potentially equivalent to an additional 5.6 human years assuming an 80-year lifespan. The enhanced survival was associated with (1) improvement in LV CO, (2) a robust reduction in systemic edema, and (3) prolonged maintenance of normal overall body composition (bodyweight, fat, and lean mass). When compared to DCM+vehicle treated animals, the DCM+DRI animals showed a significant absolute increase in EF of 5% and a relative increase of 33%. Clinical studies have shown that in patients with low EF there is a direct correlation of EF with mortality, with higher EF being linked to better survival [52]. One of the factors most correlated with mortality was ECW retention, which is linked to the onset of edema. It is interesting to note that ECW levels greater than one gram in both groups were associated with mortality, suggesting that these levels are not compatible with survival. Clinical studies investigating edema as a biomarker for mortality [53,54] support these findings; however, routine monitoring of HF patients currently does not include the non-invasive quantification of ECW, which precedes the onset of clinically detectable and symptomatic edema [55].

To our knowledge, this is the first study to evaluate the use of QMR as a modality to measure systemic edema (i.e., ECW) in a HF model. This technology provides a quick and non-invasive method to objectively quantify by weight, not image, body composition changes throughout the disease progression of HF; it identified changes in fat and lean mass associated with cardiac cachexia and sarcopenia in late stages of HF. Clinically, sarcopenia or muscle loss has been evaluated through dual-energy X-ray absorptiometry (DXA or DEXA, commonly referred to as bone mineral density scanning), magnetic resonance imaging (MRI), and computed tomography [56]. ECW is measured by MRI, bioelectrical impedance analysis, or bioimpedance spectroscopy [57]. The advantages of QMR were operational ease, recording speed, reproducibility, and accurate measurements that do not require interpretation of images [58,59,60,61]; this may prove to be a useful modality for monitoring human disease progression and the efficacy of therapy for HF.

Through vasodilation, natriuresis and other mechanisms, the NP system opposes the effects of active renin in HF. Suppression of pathological plasma renin activity concentration normalized plasma neprilysin and induced a trend towards normalization of cGMP levels, but did not affect plasma ANP and corin levels. This is notable, as elevated plasma neprilysin and cGMP levels reflect symptomatic HF, while low corin levels may reflect systolic dysfunction, independent of HF clinical symptoms (i.e., edema) in human and mice with DCM [26,27,62,63,64,65]. Elevated plasma neprilysin levels are associated with enhanced mortality in clinical HF, thus the fact that normalization of pathological plasma renin activity level reduced neprilysin levels is consistent with its effects on prolonging survival [66].

Although DRI-aliskiren at 100 mg/kg normalized pathological renin activity, there was no significant difference in plasma Ang II and aldosterone levels between DCM+vehicle and DCM+DRI groups. However, it cannot be excluded that aliskiren may suppress cardiac Ang II production as reported in diabetic rats [67]. This lower dose DRI treatment normalized plasma neprilysin levels. Lower neprilysin levels are associated with reduced degradation of NP and of angiotensin (1–7), which promotes vasodilation and sodium excretion [68]. Although active neprilysin cleaves NP, we found that there was no correlation between levels of immunoreactive NP and immunoreactive neprilysin, which has also been noted in previous studies of HF patients [69]. Additionally, aliskiren may provide cardiac protection through induction of increased cardiac bradykinin levels [50,70]. Studies also suggest that renin may modulate HF through direct stimulation of (pro)-renin signaling receptor independently from angiotensinogen-angiotensin axis [71]. However, the effects of aliskiren on local cardiac, pulmonary, and renal tissues were outside the scope of the current study. Since the present studies were designed to examine the direct pathophysiologic effects of elevated plasma renin activity in HFrEF progression, they do not provide insights into how DRI monotherapy compares to other HF therapies with or without co-administration of DRI. Further studies will be necessary to determine the generalizability of these findings to other HF conditions associated with elevated renin activity and to further explore the mechanisms through which the normalization of plasma renin activity modulates fluid-retention/edema and cachexia/sarcopenia.

In HF, cardiac cachexia is associated with increased rates of morbidity and mortality, independent of ventricular function and clinical symptoms [72]. Most experts agree that bodyweight loss of >5–6% should be used to characterize cardiac cachexia [41,56]. Serial QMR measurements permitted the detection of body fat and muscle losses, which may have been masked by simply following bodyweights as currently recommended for management of HF patients [9]. Changes in body composition started at 90 days of age and became significant at 100 days with declines in bodyweight (−8.6% compared to WT), fat composition (−45%), and lean mass (−12%) evident in the DCM+vehicle control group. Whether these changes in body composition are due to changes in metabolism or appetite is unknown, as caloric intake was not measured. The role of RAAS modulation in the alteration of sarcopenia is recognized [6,73,74]. The normalization of plasma renin activity attenuated cachexia and sarcopenia, independently of alteration of plasma Ang II levels, suggests that renin activity contributes directly or indirectly to this process.

Although there are important differences between patients, nearly everyone with HFrEF is currently treated with the same medications. These studies show that normalizing the increased plasma renin activity concentration in experimental HFrEF significantly improved systolic function, delayed the onset of edema, diminished the development of cachexia/sarcopenia, and significantly extended survival. These data support the notion that increased plasma renin activity has deleterious effects in HF and suggests that, in appropriately identified individuals, the normalization of plasma renin activity may have protective effects by delaying the transition from early, asymptomatic to more severe and fatal HF.

## 4. Materials and Methods

### 4.1. Institution and Environment

All experimental activities were reviewed and approved by the Institutional Animal Care and Use Committees at the University of Tennessee Health Science Center (15–050; approved 9 July 2015 and 17–059; approved 26 July 2017) and the University of Arizona College of Medicine – Phoenix (17–303; approved 11 December 2017). All animal activities were conducted within AAALACi accredited facilities in accordance with the National Institutes of Health (NIH) Guide for the Care and Use of Laboratory Animals. Mice were housed under a 12:12 light–dark cycle; fed an ad-lib maintenance diet (Envigo Teklad 7912; Madison, WI, USA) without consumption monitoring; in individually ventilated caging system (Optimouse, Animal Care Systems; Centennial, CO, USA) with corn cob bedding (Shepherd’s Cob+Plus, Shepherd Specialty Papers; Watertown, TN, USA).

### 4.2. Mice

The mice used for this study were randomly assigned female littermates with or without dilated cardiomyopathy (DCM) on a C57BL/6 background. DCM mice express a transgene dominant-negative CREB transcription factor specific to cardiomyocytes and consistently develop the progressive stages (A–D) of HF similar to those described for humans [22,23,24,25,26,27,75]. In female mice with DCM renal function remains within a normal range up to the terminal HF stage, as measured by plasma BUN and creatinine [27]. DCM+vehicle (*n* = 28) and DCM+DRI (*n* = 29) were compared to each other. A group of congenic WT mice (*n* = 20) was used for reference. In sub-groups of 90-day old mice (*n* = 8/group), terminal blood was collected via cardiocentesis with ethylenediaminetetraacetic acid (EDTA)-aprotinin syringes to prevent proteolysis of targeted proteins and dissected organs were weighed. The blood samples were centrifuged at 3000 rpm for 20 min at 4 °C. Plasma samples were aliquoted and stored at −80 °C until analysis. All analysis and health/death reports were recorded by investigators and animal facility technicians who were blinded to the mouse genotype.

### 4.3. Direct Renin-Inhibitor Treatment

The direct renin-inhibitor group (DCM+DRI) was administered aliskiren hemifumarate (BOC Sciences, Shirley, NY, USA) at 100 mg/kg/day orally via single source drinking-water in autoclaved hanging bottles. Dose calculations were based on an average consumption of 5 mL of water/day/mouse. The bioavailability of aliskiren is low in humans and only 2.6% orally in rats [43]. Previous studies administering 15–50 mg/kg/day via subcutaneous osmotic pumps showed no alteration in blood pressure [40,46,49,76]. Our oral dose was chosen to closely mimic the human route of delivery, while not altering the blood pressure, associated with elevated plasma Ang II–aldosterone levels, given the low bioavailability of the drug in rodents. To prepare the solution, aliskiren powder was dissolved by shaking in volume measured autoclaved 3 ppm hyperchlorinated facility water, mixed fresh every 72 h and shaken daily to resuspend. No issues with palatability or clinical dehydration were noted throughout the study as assessed by blinded husbandry and veterinary staff. There were no side effects or abnormal clinical observations in our mice throughout the treatment period. The aliskiren in drinking water was well tolerated and consumed at expected levels for plain water. The administration was started at 50 days of age to coincide with the previously identified timeline of increased renin activity and development of stage B HF in female DCM mice [27]. Aliskiren water was provided ad lib throughout life. All untreated mice (WT and DCM+vehicle) received ad lib 3 ppm hyperchlorinated facility water via an automated watering system (Edstrom, Waterford, WI, USA).

### 4.4. Body Composition

Body composition was objectively quantified by a single-blinded and experienced operator [77]. Bodyweight, free water (systemic extracellular water, ECW), total water, body fat, and lean mass were recorded longitudinally. Mice were measured every tenth day between 50–100 days using quantitative magnetic resonance (QMR) technology with an EchoMRI^TM^ 4-in-1 Analyzer (Echo Medical Systems, Houston, TX, USA). The machine was calibrated daily per standard operating procedure using the provided canola oil (54.3 g) phantom. Briefly, mice were weighed (Scout Pro SP401, Ohaus Corporation, Pine Brook, NJ, USA) and loaded into a tube restrainer specific to the system. Mice were fully conscious and minimally restrained throughout each 60–90 s recording and were returned to their home enclosure following measurement.

### 4.5. Echocardiography

The standard transthoracic exam was performed using a Vevo 2100 Imaging System (VisualSonics; Toronto, ON, Canada) with a 30 MHz transducer (MS 400) as previously reported [24,25,26,27]. All imaging was performed at 90 days of age (mice were treated for 35 days before subjecting to echocardiogram). Briefly, mice were induced with 3–5% isoflurane in oxygen and fur removed with depilatory cream (Nair, Church & Dwight Co. Inc., Princeton, NJ, USA). Maintenance anesthesia was held at 2% isoflurane in oxygen throughout the two-dimensional and M-mode recordings of the left ventricle (LV) in parasternal long-axis, short-axis, and four-chamber views. Mouse physiology was maintained at an anesthetized heart rate of 450 ± 50 beats per minute and 37 ± 1 °C rectal temperature. The analysis was blindly completed post-recording using Vevo LAB software (3.1.0, VisualSonics) with three cardiac cycles traced to produce mean values. Ejection fraction (EF, %), fractional shortening (FS, %), LV mass corrected (LVMc, mg), and cardiac output (CO, mL/min) were calculated using standard equations within the software.

### 4.6. Enzyme Immunoassay

Plasma Ang II, aldosterone, atrial natriuretic peptide (N terminus-ANP), cyclic guanosine monophosphate (cGMP), neprilysin, and corin levels were measured by enzyme immunoassays according to the manufacturers’ protocols (Phoenix Pharm. Inc., Burlingame, CA, USA; Abcam Inc., Cambridge, MA, USA; Enzo Life Sciences Inc., Farmingdale, NY, USA; Boster Biological Technology, Pleasanton, CA, USA; USCN Life Science Inc., Houston, TX, USA) as previously reported [24,25,26,27].

### 4.7. Plasma Renin Activity Assay

Renin enzymatic activity from EDTA-aprotinin supplemented mouse plasma samples (described in the Section 4.2) were measured in a 96-well microplate (Synergy HT reader and Gen5 v1.09 software, BioTek Instruments, Inc., Winooski, VT, USA) and quantified using exogenous fluorescence resonance transfer (FRET) peptide substrates of renin FRET-QXL™520/5-FAM, optimized for mouse renin (SensoLyte 520 mouse renin assay kit, AnaSpec, Fremont, CA, USA) as previously reported [25,27,32,78,79,80,81]. Cleavage of the FRET substrate by mouse renin results in the recovery of quenched fluorescence of 5-FAM, which was detected at excitation/emission = 490/520 nm with minimum autofluorescence of plasma samples. The 5-FAM fluorescent reference standard curve was used for results quantification. It is important to note that the plasma renin activity concentration assay differs from plasma renin activity (PRA) and active renin concentration (ARC)/ active plasma renin concentration (APRC) which have been historically used to report active renin in clinical trials [32].

### 4.8. Statistical Analysis

Statistical analyses were performed with GraphPad Prism 7.04 software (GraphPad Software, La Jolla, CA, USA) using Student’s *t*-test, one-way ANOVA, or two-way ANOVA with Tukey’s multiple comparisons test (unless otherwise indicated) and Pearson’s correlation. Survival was analyzed using Kaplan–Meier curves with the Mantel–Cox test. Differences were considered significant if *p* ≤ 0.05. The number of animals (*n*) is indicated in the figures or figure legends. Data are represented as mean ± SE.

## 5. Conclusions

In a translational model of DCM with rEF, the normalization of elevated plasma renin activity concentration retards the progression of experimental HF by reducing the development of systemic edema, cachexia/sarcopenia, and mortality. Normalization of elevated plasma renin activity selectively reduces neprilysin levels without affecting levels of angiotensin II and aldosterone. Further studies are warranted to determine whether the normalization of elevated plasma renin activity may be beneficial in appropriately targeted HFrEF patients.

## Abbreviations

HF	heart failure
rEF	reduced ejection fraction
DCM	renin-angiotensin-aldosterone-system
RAAS	linear dichroism
NP	natriuretic peptide
ANP	atrial natriuretic peptide
BNP	b-type natriuretic peptide
Ang II	angiotensin II
Ang (1-7)	angiotensin (1-7)
DRI	direct renin inhibitor
NEP	neprilysin
cGMP	cyclic guanosine monophosphate
ARC	active renin concentration
ELISA	enzyme-linked immunosorbent assay
AU	arbitrary units
SEM	standard error of the mean
WT	wild type
HW/BW	heart weight to bodyweight ratio
LW/BW	lung weight to bodyweight ratio
QMR	quantitative magnetic resonance
ECW	extracellular water
LV	left ventricle
LVMc	left ventricular mass corrected
PRA	plasma renin activity
ARC	active renin concentration
APRC	active plasma renin concentration
